# Patient-led, home-based follow-up for colorectal cancer: DISTANCE multicentre stepped-wedge cluster randomised trial

**DOI:** 10.1093/bjs/znag081

**Published:** 2026-06-26

**Authors:** M H Elise van Driel, Hidde Swartjes, Jobbe M G Lemmens, Seyed M Qaderi, Steven Teerenstra, Jose A E Custers, Marloes A G Elferink, Bob J van Wely, Johanne G Bloemen, Wilhelmina M U van Grevenstein, Peter van Duijvendijk, Emiel G G Verdaasdonk, Marnix A J de Roos, Veerle M H Coupé, Geraldine R Vink, Cornelis Verhoef, Dirk J Grünhagen, Johannes H W de Wilt

**Affiliations:** Department of Surgical Oncology and Gastrointestinal Surgery, Erasmus MC Cancer Institute, Rotterdam, The Netherlands; Department of Surgery, Radboud University Medical Centre, Nijmegen, The Netherlands; Department of Surgery, Radboud University Medical Centre, Nijmegen, The Netherlands; Department of Surgery, Radboud University Medical Centre, Nijmegen, The Netherlands; Department for Health Evidence, Radboud University Medical Centre, Nijmegen, The Netherlands; Department of Medical Psychology, Radboud University Medical Centre, Nijmegen, The Netherlands; Department of Research and Development, Netherlands Comprehensive Cancer Organisation, Utrecht, The Netherlands; Department of Surgery, Bernhoven Hospital, Uden, The Netherlands; Department of Surgery, Catharina Hospital, Eindhoven, The Netherlands; Department of Surgical Oncology, University Medical Centre Utrecht, Utrecht University, Utrecht, The Netherlands; Department of Surgery, Gelre Hospitals, Apeldoorn, The Netherlands; Department of Surgery, Jeroen Bosch Hospital, ‘s-Hertogenbosch, The Netherlands; Department of Surgery, Rijnstate Hospital, Arnhem, The Netherlands; Department of Epidemiology and Data Science Amsterdam Public Health, Amsterdam UMC, location Vrije Universiteit, Amsterdam, The Netherlands; Department of Medical Oncology, University Medical Centre Utrecht, Utrecht University, Utrecht, The Netherlands; Department of Surgical Oncology and Gastrointestinal Surgery, Erasmus MC Cancer Institute, Rotterdam, The Netherlands; Department of Surgical Oncology and Gastrointestinal Surgery, Erasmus MC Cancer Institute, Rotterdam, The Netherlands; Department of Surgery, Radboud University Medical Centre, Nijmegen, The Netherlands

## Abstract

**Background:**

Colorectal cancer (CRC) incidence is rising; consequently, traditional follow-up care models are increasingly unsustainable. Patient-led, home-based follow-up (PHFU) may offer a promising alternative to reduce hospital visits while maintaining patient well-being.

**Methods:**

The DISTANCE trial was a stepped-wedge cluster randomised trial conducted in six hospitals in the Netherlands. A total of 354 stage I–III CRC survivors, disease-free at 12 months after surgery, were assigned to either PHFU or standard follow-up. The primary endpoint was the number of hospital contacts. Secondary endpoints included quality of life (assessed using the European Organisation for Research and Treatment of Cancer core quality-of-life questionnaire (EORTC QLQ-C30); version 3.0), cancer-related worry (assessed using the Cancer Worry Scale (CWS)), and psychological distress (assessed using the Hospital Anxiety and Depression Scale (HADS)).

**Results:**

In the intention-to-treat (ITT) analysis, no significant difference in hospital contacts was observed. In the as-treated (AT) analysis, PHFU reduced hospital contacts by 38% compared with standard follow-up (rate ratio 0.62 (95% c.i. 0.51 to 0.75), *P* < 0.001). There was substantial crossover, with 117 patients assigned to PHFU receiving standard follow-up and 10 patients assigned to standard follow-up receiving PHFU. No significant differences between the two groups were found with regard to quality of life or psychological well-being.

**Conclusion:**

The DISTANCE trial suggests that PHFU is a feasible and effective alternative to standard hospital-based follow-up for CRC survivors.

## Teaser

In this multicentre stepped-wedge cluster randomised trial, patient-led, home-based follow-up (PHFU) for colorectal cancer (CRC) survivors was evaluated in routine surgical practice. PHFU reduced hospital follow-up contacts by 38% in the as-treated (AT) analysis, without compromising quality of life or psychological well-being. Substantial crossover between allocated and delivered care highlighted that implementation, rather than patient acceptance, was the main barrier to reducing follow-up intensity.

## Introduction

Colorectal cancer (CRC) is one of the most common malignancies. Owing to an ageing population and lifestyle-related risk factors, the global incidence of CRC has risen, and projections suggest that, by 2040, the number of CRC cases will reach 3.2 million, a 63% increase^1^. This growing patient population, combined with a relatively shrinking healthcare workforce, poses major challenges for the sustainability of follow-up care. At the same time, advances in early detection and surgical techniques have improved survival. Nevertheless, approximately 30% of patients with stage I–III CRC experience recurrence or metastasis within 5 years after curative treatment^2–4^. Early detection of such recurrences is thought to allow timely interventions and potentially improve survival outcomes, underscoring the need for effective and efficient follow-up strategies.

Recent evidence, however, challenges the effectiveness of intensive follow-up. Both the 2022 systematic review by Galjart *et al*.^5^ and the FACS RCT^6^ showed that intensified follow-up, while leading to more treatments, does not improve survival. In addition, early detection of recurrence may impose an unnecessary psychological and physical burden on patients^7^. These findings highlight the need to reconsider current follow-up strategies and to explore alternative models that can achieve comparable or improved outcomes while using fewer healthcare resources^8^.

In response to these challenges, patient-centred, home-based follow-up models have emerged as promising alternatives. Such approaches aim to empower patients to take a more active role in their care, potentially enhancing satisfaction while reducing the burden on healthcare systems. A single-centre study by Qaderi *et al*.^9,10^ demonstrated that a remote follow-up plan for curatively treated, non-metastatic CRC patients was feasible, cost-effective, and associated with a comparable quality of life to traditional hospital-based follow-up. Decisive evidence is still lacking, as no multicentre trial has yet been conducted.

The aims of the DISTANCE trial were to evaluate whether a patient-led, home-based follow-up (PHFU) model could be successfully implemented as a new follow-up strategy and reduce the number of contact moments between patient and hospital and to evaluate patient-reported outcomes of this model in comparison with standard hospital-based follow-up.

## Methods

The DISTANCE trial was a stepped-wedge cluster randomised trial, the full protocol of which has been published previously^11^. It was conducted in six hospitals in the south and east of the Netherlands. The cluster units were the hospitals, which were randomised to sequences determining the timing of crossover from standard follow-up to PHFU. The allocation of transition moments was randomised before the start of the study using a live, online randomisation process with a computer-generated die, ensuring transparency and fairness to all local investigators. The intended implementation schedule and the actual timing of implementation are shown in *Appendix SB* and *Appendix SA* respectively. The actual implementation deviated slightly due to logistical challenges and delays in approval at certain centres.

The study’s observation interval spanned from 12 to 24 months after curative CRC surgery. Individual patients were enrolled once, at 12 months after surgery, and were then followed for 12 months; thus, different patients could be included in different calendar intervals. This study is reported in accordance with the CONSORT extension for stepped-wedge cluster randomised trials^12^.

### Ethics approval and consent to participate

This study was performed in line with the principles of the Declaration of Helsinki. According to the Medical Research Ethics Committee Oost Nederland, the present study (file number: 2020-7105) was not subject to the Medical Research Involving Human Subjects Act (WMO), and the study was approved by all local institutional review boards. All patients provided written approval for study participation. The DISTANCE trial is registered in the Dutch Trial Register (NTR; NL9266).

### Study population

This study built upon the infrastructure of the Prospective Dutch CRC cohort (PLCRC)^13^. While the PLCRC consists of all patients diagnosed with CRC who have provided broad informed consent for the use of medical data for scientific research, the DISTANCE trial focused on a more specific subgroup. Eligible clusters were hospitals participating in the DISTANCE implementation study and able to deliver both standard follow-up and PHFU according to the study protocol. Eligible participants were ≥18 years and disease-free at 12 months (based on CT) after curative resection for stage pT2–4 N0 M0 or pT1–4 N1–2 M0 CRC. Exclusion criteria were macroscopically incomplete resection (R2), a need for extended hospital care due to a complicated recovery or medical co-morbidities, or hereditary CRC. A total of 360 patients were needed based on the sample size calculation^11^.

### Intervention and control

Before implementation of the PHFU protocol, patients received standard in-hospital follow-up in accordance with national CRC guidelines, including scheduled determination of blood carcinoembryonic antigen (CEA) levels and imaging^14^. In the phase of allocating patients to PHFU, after additional verbal consent, patients followed the same schedule but with home-based determination of blood CEA levels at local facilities (for example general practitioner surgeries). Blood results were provided digitally; physician contact occurred only if CEA levels were elevated, if initiated by the patient, or, if requested by the treating physician.

### Outcome measures

The primary endpoint was defined as the number of contact moments with the hospital for follow-up care between 12 and 24 months after curative surgical resection. This was compared between the two follow-up models: in-hospital standard care and PHFU.

Secondary endpoints included: quality of life, assessed using the European Organisation for Research and Treatment of Cancer core quality-of-life questionnaire (EORTC QLQ-C30; version 3.0); fear of cancer recurrence, assessed using the Cancer Worry Scale (CWS); and anxiety, depression, and distress, assessed using the Hospital Anxiety and Depression Scale (HADS).

### Data collection

Data were obtained from the Netherlands Cancer Registry (NCR), the PLCRC database, and hospital electronic records.

### Statistical analysis

Primary outcome analyses followed both the intention-to-treat (ITT) principle, whereby patients were analysed according to the follow-up strategy assigned to their hospital at the time of inclusion, based on the actual implementation status of the stepped-wedge design (standard *versus* PHFU), and an as-treated (AT) approach, based on the follow-up actually received, without excluding patients who did not receive their originally assigned follow-up strategy.

The primary outcome, defined as the mean contact rate (that is the mean number of contact moments per month), was analysed using generalised linear mixed models (GLMMs) with a Poisson distribution and log link. A random intercept for hospital accounted for within-hospital clustering, and fixed effects included treatment group and recruitment interval (calendar quarter) to adjust for temporal trends. To account for varying follow-up durations, the natural logarithm of follow-up time (in months) was included as an offset, yielding rate ratios (RRs) per month. Model fit was assessed using residual diagnostics and comparison with a negative binomial model. Based on model fit criteria (Akaike Information Criterion (AIC)), the Poisson model provided a better fit to the data and was therefore used for the final analyses. Results are reported as RRs with 95% confidence intervals and *P* values. All *P* values are two-sided and *P* < 0.050 was considered statistically significant.

Quality of life was assessed using version 3.0 of the EORTC QLQ-C30 at 12, 18, and 24 months after surgery. Scales and single items were scored according to the EORTC QLQ-C30 scoring manual and linearly transformed to a 0–100 scale (with higher scores indicating better functioning and worse symptoms). In line with recent recommendations, the EORTC QLQ-C30 summary score was calculated as the mean of 13 functioning and symptom scales, excluding Global Health Status and Financial Difficulties^15^. Before averaging, all symptom scales were reversed so that higher values consistently reflected better quality of life. Cancer-related worry was assessed using the six-item CWS; the total score was calculated as the sum of the six items. Anxiety and depression were assessed using the HADS; subscale scores (HADS-anxiety and HADS-depression) were calculated as the sum of their respective seven items if five or more items were completed. The total distress score (HADS-distress) was derived by summing the HADS-anxiety and HADS-depression scores. For both the CWS and HADS scores, higher values indicated greater worry, anxiety, depression, or distress.

For each EORTC QLQ-C30 scale, the EORTC QLQ-C30 summary score, the CWS, and the HADS subscales, descriptive statistics (mean(s.d.) values) were calculated for each study group and time point. Differences between groups were analysed using separate linear mixed-effects models with a random intercept for patient and fixed effects for hospital, follow-up group, time point, and their interaction. *P* values for main and interaction effects were derived using Satterthwaite’s approximation. To account for multiple testing across all scales, Holm correction was applied.

Baseline characteristics are presented as *n* (%) for categorical variables and mean (s.d.) for continuous variables. Group comparability was assessed using standardised differences (SMDs), with values >0.1 considered indicative of meaningful imbalance. Missing data were excluded from the analysis where applicable. Statistical analysis was performed in R (version 4.4.2).

## Results

### Study population

**Figure 1:**
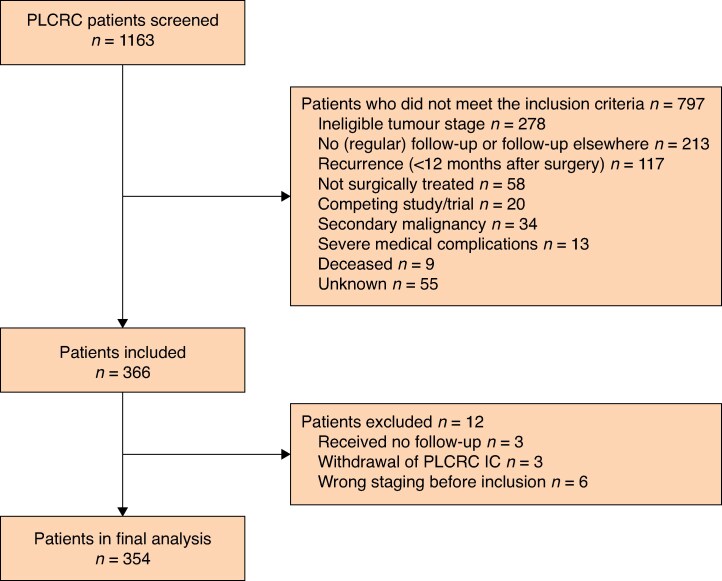
Flow chart illustrating the inclusion/exclusion process PLCRC, Prospective Dutch CRC cohort; IC, informed consent.

A total of 1163 patients who had provided informed consent for participation in the PLCRC were screened for eligibility. Of these patients, 797 did not meet the inclusion criteria; the specific reasons and corresponding numbers are presented in *Fig. 1*. An overview of screening for each hospital can be found in *Appendix SC*. After the initial screening, 366 patients were enrolled. During the final analysis, 12 additional patients were excluded post hoc (with reasons also detailed in *Fig. 1*), yielding a final study population of 354 patients (included between July 2022 and May 2024).

Baseline characteristics for both the ITT population and the AT population are presented in *Table 1*. In the ITT cohort, 175 patients received standard follow-up and 179 patients received PHFU; in the AT cohort, 282 patients received standard follow-up and 72 patients received PHFU. There was considerable crossover between allocated and received follow-up: 10 patients allocated to the control arm received PHFU, whereas 117 patients allocated to PHFU ultimately received standard follow-up.

**Table 1:** Demographic and tumour characteristics at baseline for standard follow-up and PHFU (ITT and AT)

	ITT			AT		
	Standard follow-up (*n* = 175)	PHFU (*n* = 179)	SMD	Standard follow-up (*n* = 282)	PHFU (*n* = 72)	SMD
**Sex**						
Male	95 (54)	105 (59)	0.088	157 (56)	43 (60)	0.082
Female	80 (46)	74 (41)		125 (44)	29 (40)	
Age (years), mean(s.d.)	66.8(10)	68.1(9)	0.135	67.71(10)	66.35(7)	0.155
**Co-morbidities**						
No co-morbidities	88 (64)	78 (63)	0.175	131 (62)	35 (70)	0.165
One co-morbidity	38 (28)	29 (24)		56 (27)	11 (22)	
At least two co-morbidities	11 (8)	16 (13)		23 (11)	4 (8)	
**Location of primary tumour**						
Right hemi-colon	67 (38)	57 (32)	0.231	99 (35)	25 (35)	0.152
Transverse colon	9 (5)	15 (8)		20 (7)	4 (6)	
Left hemi-colon	55 (31)	70 (39)		96 (34)	29 (40)	
Rectum	44 (25)	37 (21)		67 (24)	14 (19)	
**(y)pT category**						
0	4 (2)	1 (1)	0.222	5 (2)	0 (0)	0.259
1	2 (1)	6 (3)		6 (2)	2 (3)	
2	56 (32)	59 (33)		89 (32)	26 (37)	
3	102 (58)	98 (55)		160 (57)	40 (56)	
4	11 (6)	14 (8)		22 (8)	3 (4)	
**(y)pN category**						
X/0	117 (67)	125 (70)	0.159	192 (68)	50 (70)	0.188
1	53 (30)	44 (25)		77 (27)	20 (28)	
2	5 (3)	9 (5)		13 (5)	1 (1)	
**Radiotherapy**						
No radiotherapy	156 (89)	157 (88)	0.579	246 (87)	67 (93)	2.132
Chemoradiation	13 (7)	11 (6)		24 (9)	0 (0)	
Short-course radiotherapy	5 (3)	11 (6)		11 (4)	5 (7)	
Long-course radiotherapy	1 (1)	0 (0)		1 (0)	0 (0)	
**Chemotherapy**						
No chemotherapy	118 (67)	125 (70)	0.284	186 (66)	57 (79)	0.423
Preoperative chemotherapy	15 (9)	12 (7)		26 (9)	1 (1)	
Postoperative chemotherapy	42 (24)	36 (20)		66 (23)	12 (17)	
Preoperative and postoperative chemotherapy	0 (0)	6 (3)		4 (1)	2 (3)	
**Colon carcinoma**						
N−/Nx	85 (65)	100 (70)	0.119	144 (67)	41 (71)	0.080
N+	46 (35)	42 (30)		71 (33)	17 (29)	
**Rectum carcinoma**						
N−/Nx	32 (73)	25 (69)	0.072	48 (72)	9 (69)	0.053
N+	12 (27)	11 (31)		19 (28)	4 (31)	

Values are *n* (%) unless otherwise indicated. PHFU, patient-led, home-based follow-up; ITT, intention-to-treat; AT, as-treated; SMD, standardised mean difference.

Throughout the study interval, 12–24 months after surgery, 13 patients (3.7%) experienced disease recurrence. In the ITT analysis, seven recurrences occurred in the group that received standard follow-up (175 patients) and six recurrences occurred in the group that received PHFU (179 patients). In the AT analysis, 12 recurrences were observed in the group that received standard follow-up (282 patients) and one recurrence occurred in the group that received PHFU (72 patients). In addition, two patients (0.6%) died, one of whom died as a consequence of recurrent disease. Both of these patients were retained in the final analysis.

Baseline characteristics were generally well balanced between groups in the ITT analysis. In the AT analysis, some imbalances were observed, particularly for treatment-related variables such as radiotherapy and chemotherapy. These imbalances may reflect non-adjustment for time trends, clustering, or residual confounding.

*Table 2* shows the distribution of participants per follow-up group according to the ITT and AT populations, stratified by hospital. Deviations from the allocated follow-up method were observed in all centres.

**Table 2:** Number of participants per follow-up group (ITT *versus* AT) by centre

Centre*	ITT		AT		Adherence to protocol (%)
	Standard follow-up	PHFU	Standard follow-up	PHFU	
1	36	39	57	18	31.6
2	28	55	67	16	29.1
3	11	35	32	14	40.0
4	50	15	52	13	86.7
5	29	11	37	3	27.3
6	21	24	37	8	33.3

^*^Participating centres are listed in a random order. ITT, intention-to-treat; AT, as-treated; PHFU, patient-led, home-based follow-up.

### Primary endpoint (number of contact moments)

In the ITT analysis, the mean contact rate did not differ significantly between patients assigned to PHFU and patients assigned to standard follow-up (RR 1.09 (95% c.i. 0.86 to 1.37), *P* = 0.48). None of the recruitment interval coefficients was different (all *P* > 0.14), indicating no relevant temporal trend in contact frequency over the study interval. The estimated mean(s.d.) variance of the hospital-level random intercept was 0.078(0.278).

In the AT analysis, the mean contact rate differed between groups. Patients who received PHFU had a 38% lower follow-up contact rate compared with patients who received standard hospital-based follow-up (RR 0.62 (95% c.i. 0.51 to 0.75), *P* < 0.001). Comparable to the ITT analysis, recruitment interval effects were not statistically significant, indicating no temporal trend in follow-up rates. Modest hospital-level clustering was present (mean(s.d.) variance of 0.070(0.265)), suggesting some variation between hospitals. Residual diagnostics indicated no evidence of model misfit or overdispersion, confirming the appropriateness of the model.

### Different types of hospital contact

Across all participating centres, telephone consultations represented the majority of follow-up contacts in both the standard follow-up group and the PHFU group in the AT analysis (*Table 3*), whereas outpatient clinic visits accounted for a smaller proportion. Video consultations and digital messages were infrequently used across either group. The overall pattern was consistent across centres, with only minor variation. Missing data were minimal.

**Table 3:** Breakdown of modalities for contact moments across participating centres

Centre	Type of follow-up	Total number of patients	Total number of contact moments	Outpatient clinic visits	Video consultations	Telephone consultations	Digital messages	Missing
1	Standard follow-up	57	124	29 (23)	0 (0)	91 (73)	2 (2)	2 (2)
	PHFU	18	21	2 (9.5)	0 (0.0)	18 (85.7)	1 (4.8)	0 (0.0)
2	Standard follow-up	67	239	31 (13)	0 (0)	206 (86)	0 (0)	2 (1)
	PHFU	16	54	12 (22.2)	0 (0.0)	41 (75.9)	0 (0.0)	1 (1.9)
3	Standard follow-up	32	87	17 (19.5)	0 (0.0)	69 (79.3)	0 (0.0)	1 (1.2)
	PHFU	14	20	1 (5.0)	0 (0.0)	19 (95.0)	0 (0.0)	0 (0.0)
4	Standard follow-up	52	237	21 (9)	0 (0)	215 (91)	1 (0)	0 (0)
	PHFU	13	32	1 (3.1)	0 (0.0)	31 (96.9)	0 (0.0)	0 (0.0)
5	Standard follow-up	37	127	25 (20)	0 (0)	101 (80)	1 (1)	0 (0)
	PHFU	3	4	1 (25.0)	0 (0.0)	3 (75.0)	0 (0.0)	0 (0.0)
6	Standard follow-up	37	99	19 (19.2)	4 (4.0)	74 (74.8)	0 (0.0)	2 (2.0)
	PHFU	8	13	3 (23.1)	0 (0.0)	10 (76.9)	0 (0.0)	0 (0.0)

Values are *n* (%) unless otherwise indicated. Percentages are calculated within each hospital and follow-up group. Only AT groups are presented; due to substantial crossover between groups in the ITT population, those data were not further analysed. PHFU, patient-led, home-based follow-up; AT, as-treated; ITT, intention-to-treat.

### Quality of life

*Table 4* presents the mean(s.d,) scores for EORTC QLQ-C30 functional and symptom scales, CWS, and HADS at 12, 18, and 24 months after surgery in the AT population. Overall quality of life was high in both groups, with functional scores around 81–91 and a low symptom burden across time points. CWS scores remained stable, indicating low cancer-related worry, and HADS scores reflected low levels of anxiety and depression. No consistent differences were observed between standard follow-up and PHFU. In unadjusted analyses, no differences were found over time, except for appetite loss (*P* = 0.027), which was not significant after correction for multiple testing.

**Table 4:** EORTC QLQ-C30, CWS, and HADS scores by follow-up group at 12, 18, and 24 months

	Standard follow-up			PHFU			*P*	*P* interaction
	12m (baseline)	18m	24m	12m (baseline)	18m	24m		
EORTC QLQ-C30 functional scales (scale 0–100)*								
Summary score	88.8(9.6)	89.0(9.9)	88.9(9.7)	90.2(8.3)	90.0(8.1)	90.7(8.0)	0.468	0.820
Physical	88.7(14.9)	88.0(15.0)	88.1(14.9)	88.0(14.4)	88.7(13.7)	90.9(11.9)	0.621	0.281
Role	87.2(19.0)	85.4(20.6)	86.7(20.9)	85.7(19.1)	83.3(24.3)	87.1(17.6)	0.501	0.854
Emotional	87.2(15.7)	88.0(16.8)	88.9(15.6)	87.7(14.5)	88.3(13.7)	88.8(15.2)	0.837	0.773
Cognitive	88.4(15.4)	86.5(17.2)	88.1(14.2)	88.7(16.3)	90.8(13.2)	91.3(13.2)	0.171	0.292
Social	89.1(16.8)	89.6(16.7)	90.7(17.7)	91.3(14.4)	90.1(19.2)	90.5(16.2)	0.890	0.659
**EORTC QLQ-C30 symptom scales (scale 0–100)†**								
Fatigue	19.2(18.7)	18.2(19.0)	19.2(18.9)	21.1(18.9)	16.8(16.1)	15.8(17.1)	0.711	0.262
Nausea	2.3(9.6)	3.2(9.6)	2.4(10.1)	1.7(6.1)	1.0(4.0)	2.3(6.8)	0.258	0.575
Pain	9.8(17.6)	11.4(19.8)	13.0(20.7)	7.3(15.1)	12.2(20.6)	12.9(17.6)	0.927	0.834
Dyspnoea	11.0(18.1)	8.9(15.7)	11.0(19.8)	10.0(18.1)	11.6(18.7)	6.1(13.0)	0.924	0.034
Insomnia	22.0(26.5)	19.0(24.5)	20.5(24.5)	18.7(21.5)	19.0(21.5)	19.7(23.1)	0.650	0.492
Appetite	4.5(13.6)	3.8(13.6)	3.3(11.5)	1.3(6.6)	0.0(0.0)	1.5(7.0)	0.027	0.654
Constipation	6.9(15.4)	9.1(18.3)	9.5(16.1)	6.0(14.6)	8.2(17.4)	6.8(15.4)	0.398	0.829
Diarrhoea	10.8(18.4)	8.0(16.2)	8.6(18.0)	4.7(11.7)	4.8(11.8)	7.6(15.9)	0.102	0.269
Financial	2.4(10.1)	3.0(10.9)	3.1(12.6)	2.0(8.0)	0.7(4.8)	2.3(8.5)	0.343	0.358
**CWS (scale 6–24)**								
Score	8.1(4.2)	7.9(3.5)	7.5(4.0)	8.2(3.9)	8.3(3.2)	8.2(3.0)	0.577	0.958
**HADS (scale 0–21)**								
Anxiety	2.9(3.3)	3.2(3.2)	3.0(3.5)	2.6(2.7)	2.9(3.3)	2.7(2.7)	0.527	0.830
Depression	2.7(2.9)	2.7(2.9)	2.6(2.7)	2.4(2.5)	2.5(2.5)	2.4(2.0)	0.895	0.732
Distress	5.7(5.8)	5.9(5.7)	5.7(5.7)	5.2(4.5)	5.4(5.1)	5.0(4.0)	0.633	0.814

Values are mean(s.d.) unless otherwise indicated. *P* values refer to between-group differences (main effect of group) and the group-by-time interaction from linear mixed-effects models including a random intercept for patient and fixed effects for hospital, group, time, and their interaction. *For the summary score and functional scales, high scores indicate good functionality and low scores indicate poor functionality. †For symptom scales, high scores indicate a high symptom burden and low scores indicate a low symptom burden. EORTC QLQ-C30, European Organisation for Research and Treatment of Cancer core quality-of-life questionnaire; CWS, Cancer Worry Scale; HADS, Hospital Anxiety and Depression Scale; PHFU, patient-led, home-based follow-up.

## Discussion

This multicentre stepped-wedge cluster randomised trial evaluated the implementation of PHFU for CRC survivors in routine practice across six Dutch hospitals. While PHFU did not reduce hospital contacts in the ITT analysis, a 38% reduction was observed in the AT analysis, without adverse effects on quality of life or psychological well-being.

Baseline characteristics were generally well balanced in the ITT analysis. Imbalances in treatment-related variables were observed in the AT analysis but were not considered clinically relevant, as prior treatments were completed before enrolment and did not require more intensive follow-up. These differences likely reflect residual confounding or unadjusted time trends and clustering.

Introducing home-based follow-up proved challenging across centres. The large discrepancy between ITT and AT results underscores the challenge of fully implementing new care models in real-world settings. The high crossover rate was primarily driven by incomplete implementation of the PHFU strategy at the clinician level. In many cases, PHFU was not consistently offered or discussed during follow-up visits. This highlights that clinician engagement and institutional routines, rather than patient willingness, are likely key determinants of successful implementation. Previous work has shown that leadership and staff buy-in are decisive for the adoption of innovative care pathways, with clinician behaviour being the main driver of change^16,17^. These challenges apply not only to the implementation of a new follow-up strategy but also to its evaluation within a stepped-wedge design.

The centre-specific effects observed in this study highlight that adherence to the PHFU protocol was strongly influenced by local practice patterns, with each centre’s routines playing a decisive role. In some centres, follow-up was primarily coordinated by clinicians, whereas, in others, it was managed by specialised nurses or physician assistants. This variation reflects the known differences in follow-up organisation across Dutch hospitals. Previous national studies have shown that both treatment strategies and oncological outcomes vary across hospital types and volumes^18,19^.

The low number of recurrences during the 12–24-month study window likely reflects the declining recurrence risk beyond the first postoperative year. Previous studies have shown that most CRC recurrences occur within the first 2 years after surgery, after which rates decrease sharply^20^. This supports the safety of PHFU during this later phase of surveillance. In the AT analysis, recurrence rates were numerically higher in the standard follow-up group, not accounting for clustering and time trend. However, given the small number of events and the non-adjusted analysis, this difference is most likely due to chance or residual confounding. Importantly, the study was not powered or type I error controlled to detect differences in recurrence rates.

Quality of life remained high throughout follow-up, consistent with prior studies showing that less intensive surveillance does not compromise health-related quality of life, emotional well-being, or patient satisfaction^9,21,22^. These stable outcomes suggest that PHFU maintains patient well-being while reducing the need for in-hospital visits.

Several limitations should be considered. The divergence between ITT and AT outcomes reflects the inherent challenges of achieving full adherence in pragmatic designs. While a pragmatic design facilitates efficient recruitment and enhances external validity, it limits control over implementation, as treating physicians determine whether and how to introduce the intervention. This contributed to crossover from PHFU to standard follow-up. It was often not documented whether or not PHFU was discussed during the inclusion visit, which could introduce possible unmeasured selection bias. Furthermore, the follow-up interval was limited to 12–24 months after surgery, preventing evaluation of long-term outcomes such as late recurrences or sustained quality-of-life changes. Finally, inclusion was restricted to PLCRC participants who were disease-free at 12 months, which may have selected a healthier and more engaged survivor population.

The results of the DISTANCE trial suggest that PHFU is a promising model for CRC survivor care. The principal challenge is not the safety or acceptability of PHFU for patients but its consistent implementation in everyday practice. To fully realise its potential, PHFU needs to be better integrated into clinical workflows, with stronger clinician engagement and tailored implementation strategies across centres. Overcoming these barriers will be key to scaling PHFU effectively.

As the incidence of CRC continues to rise, sustainable follow-up strategies are increasingly important. PHFU offers a promising way to reduce unnecessary hospital visits while maintaining patient safety. The present study shows that PHFU can lower follow-up contact rates without compromising quality of care. Similarly, the FUTURE-primary study demonstrated that patient-tailored, less intensive follow-up after curative surgery is safe and feasible^23^. Together, these findings suggest that follow-up need not be a one-size-fits-all approach but can be personalised to balance safety, patient preferences, and efficient resource use. Future work should focus on strategies to further enhance clinician adoption, such as integration into electronic health records, structured training, and decision-support tools. Long-term analyses of cost-effectiveness, to be reported separately, will further clarify the potential role of PHFU in national guidelines and inform its scalability to other cancer populations.

## Supplementary Material

znag081_Supplementary_Data

## Data Availability

Coded patient data and related documents (that is the study protocol and the Dutch informed consent form) are available upon reasonable request and after approval of an analysis proposal. Requests should be sent to the last author (J.H.W.d.W.).

## References

[1] Morgan E, Arnold M, Gini A, Lorenzoni V, Cabasag CJ, Laversanne M et al Global burden of colorectal cancer in 2020 and 2040: incidence and mortality estimates from GLOBOCAN. Gut 2023;72:338–344 36604116 10.1136/gutjnl-2022-327736

[2] van der Stok EP, Spaander MCW, Grünhagen DJ, Verhoef C, Kuipers EJ. Surveillance after curative treatment for colorectal cancer. Nat Rev Clin Oncol 2017;14:297–315 27995949 10.1038/nrclinonc.2016.199

[3] Qaderi SM, Galjart B, Verhoef C, Slooter GD, Koopman M, Verhoeven RHA et al Disease recurrence after colorectal cancer surgery in the modern era: a population-based study. Int J Colorectal Dis 2021;36:2399–2410 33813606 10.1007/s00384-021-03914-w PMC8505312

[4] Boute TC, Swartjes H, Greuter MJE, Elferink MAG, van Eekelen R, Vink GR et al Cumulative incidence, risk factors, and overall survival of disease recurrence after curative resection of stage II-III colorectal cancer: a population-based study. Cancer Res Commun 2024;4:607–616 38363145 10.1158/2767-9764.CRC-23-0512 PMC10903299

[5] Galjart B, Hoppener DJ, Aerts J, Bangma CH, Verhoef C, Grunhagen DJ. Follow-up strategy and survival for five common cancers: a meta-analysis. Eur J Cancer 2022;174:185–199 36037595 10.1016/j.ejca.2022.07.025

[6] Primrose JN, Perera R, Gray A, Rose P, Fuller A, Corkhill A et al Effect of 3 to 5 years of scheduled CEA and CT follow-up to detect recurrence of colorectal cancer: the FACS randomized clinical trial. JAMA 2014;311:263–270 24430319 10.1001/jama.2013.285718

[7] Welch HG, Dossett LA. Routine surveillance for cancer metastases—does it help or harm patients? N Engl J Med 2025;392:1667–1670 40293173 10.1056/NEJMp2414159

[8] Qaderi SM, Swartjes H, Custers JAE, de Wilt JHW. Health care provider and patient preparedness for alternative colorectal cancer follow-up; a review. Eur J Surg Oncol 2020;46:1779–1788 32571636 10.1016/j.ejso.2020.06.017

[9] Qaderi SM, Swartjes H, Vromen H, Bremers AJA, Custers JAE, de Wilt JHW. Acceptability, quality of life and cost overview of a remote follow-up plan for patients with colorectal cancer. Eur J Surg Oncol 2021;47:1637–1644 33423826 10.1016/j.ejso.2020.12.018

[10] Qaderi SM, Vromen H, Dekker HM, Stommel MWJ, Bremers AJA, de Wilt JHW. Development and implementation of a remote follow-up plan for colorectal cancer patients. Eur J Surg Oncol 2020;46:429–432 31668976 10.1016/j.ejso.2019.10.014

[11] Swartjes H, Qaderi SM, Teerenstra S, Custers JAE, Elferink MAG, van Wely BJ et al Towards patient-led follow-up after curative surgical resection of stage I, II and III colorectal cancer (DISTANCE-trial): a study protocol for a stepped-wedge cluster-randomised trial. BMC Cancer 2023;23:838 37679735 10.1186/s12885-023-11297-0 PMC10483744

[12] Hemming K, Taljaard M, McKenzie JE, Hooper R, Copas A, Thompson JA et al Reporting of stepped wedge cluster randomised trials: extension of the CONSORT 2010 statement with explanation and elaboration. BMJ 2018:k1614. DOI: 10.1136/bmj.k1614 30413417 PMC6225589

[13] Burbach JPM, Kurk SA, Coebergh van den Braak RRJ, Dik VK, May AM, Meijer GA et al Prospective Dutch colorectal cancer cohort: an infrastructure for long-term observational, prognostic, predictive and (randomized) intervention research. Acta Oncol 2016;55:1273–1280 27560599 10.1080/0284186X.2016.1189094

[14] *Federatie Medisch Specialisten* . *Follow-up na oncologische resectie CC en RC*. https://richtlijnendatabase.nl/richtlijn/colorectaal_carcinoom_crc/follow-up_bij_crc/follow-up_na_chirurgische_resectie_stadium_i-iii_colon-_en_rectumcarcinoom.html (accessed 13 February 2024)

[15] Giesinger JM, Kieffer JM, Fayers PM, Groenvold M, Petersen MA, Scott NW et al Replication and validation of higher order models demonstrated that a summary score for the EORTC QLQ-C30 is robust. J Clin Epidemiol 2016;69:79–88 26327487 10.1016/j.jclinepi.2015.08.007

[16] Alfano CM, Oeffinger K, Sanft T, Tortorella B. Engaging TEAM medicine in patient care: redefining cancer survivorship from diagnosis. Am Soc Clin Oncol Educ Book 2022;42:1–11 10.1200/EDBK_349391 35649204

[17] Forsythe LP, Carman KL, Szydlowski V, Fayish L, Davidson L, Hickam DH et al Patient engagement in research: early findings from the Patient-Centered Outcomes Research Institute. Health Aff (Millwood) 2019;38:359–367 30830822 10.1377/hlthaff.2018.05067

[18] Elferink MA, Krijnen P, Wouters MW, Lemmens VE, Jansen-Landheer ML, van de Velde CJ et al Variation in treatment and outcome of patients with rectal cancer by region, hospital type and volume in the Netherlands. Eur J Surg Oncol 2010; 36(Suppl 1): S74–S82 20598844 10.1016/j.ejso.2010.06.028

[19] Boute TC, van Eekelen R, Elferink MAG, LWs B, de Wilt JHW, Vink GR et al Follow-up adherence after treatment with curative intent for stage II and III colorectal cancer patients. Cancer Med 2025;14:e70667 40013322 10.1002/cam4.70667 PMC11865713

[20] Qaderi SM, Dickman PW, de Wilt JHW, Verhoeven RHA. Conditional survival and cure of patients with colon or rectal cancer: a population-based study. J Natl Compr Canc Netw 2020;18:1230–1237 32886900 10.6004/jnccn.2020.7568

[21] Wullaert L, Voigt KR, Verhoef C, Husson O, Grunhagen DJ. Oncological surgery follow-up and quality of life: meta-analysis. Br J Surg 2023;110:655–665 36781387 10.1093/bjs/znad022 PMC10364539

[22] Qaderi SM, van der Heijden JAG, Verhoeven RHA, de Wilt JHW, Custers JAE, group P. Trajectories of health-related quality of life and psychological distress in patients with colorectal cancer: a population-based study. Eur J Cancer 2021;158:144–155 34666216 10.1016/j.ejca.2021.08.050

[23] van Driel MHE, Wullaert L, Voigt KR, Outmani L, Doornebosch PG, Peeters K et al Patient-led follow-up after curative colorectal cancer surgery: the primary outcome analysis of the prospective, multicentre FUTURE-primary implementation study. Eur J Cancer 2025;226:115599 40712408 10.1016/j.ejca.2025.115599

