# Supplementary material for: Patient-led, home-based follow-up for colorectal cancer: DISTANCE multicentre stepped-wedge cluster randomised trial
Source: Br J Surg. 2026 Jun 26;113(8):znag081. doi: 10.1093/bjs/znag081 (PMC13454712; doi:10.1093/bjs/znag081)
Supplement: Supplementary file 1 [file znag081_supplementary_data.zip › Supplementary_material.docx]

**Patient-led, home-based follow-up for colorectal cancer: the DISTANCE multicentre stepped-wedge cluster-randomised trial**

M.H. Elise van Driel^1^, [Hidde Swartjes](https://pubmed.ncbi.nlm.nih.gov/?term=Swartjes+H&cauthor_id=37679735)^2^, Jobbe M.G. Lemmens^2^, [Seyed M. Qaderi](https://pubmed.ncbi.nlm.nih.gov/?term=Qaderi+SM&cauthor_id=37679735)^2^, [Steven Teerenstra](https://pubmed.ncbi.nlm.nih.gov/?term=Teerenstra+S&cauthor_id=37679735)^3^, [Jose A.E. Custers](https://pubmed.ncbi.nlm.nih.gov/?term=Custers+JAE&cauthor_id=37679735)^4^, [Marloes A.G. Elferink](https://pubmed.ncbi.nlm.nih.gov/?term=Elferink+MAG&cauthor_id=37679735)^5^, [Bob J. van Wely](https://pubmed.ncbi.nlm.nih.gov/?term=van+Wely+BJ&cauthor_id=37679735)^6^, Johanne G. Bloemen^7^, [Wilhelmina M.U. van Grevenstein](https://pubmed.ncbi.nlm.nih.gov/?term=van+Grevenstein+WMU&cauthor_id=37679735)^8^, [Peter van Duijvendijk](https://pubmed.ncbi.nlm.nih.gov/?term=van+Duijvendijk+P&cauthor_id=37679735)^9^, [Emiel G.G. Verdaasdonk](https://pubmed.ncbi.nlm.nih.gov/?term=Verdaasdonk+EGG&cauthor_id=37679735)^10^, [Marnix A. J. de Roos](https://pubmed.ncbi.nlm.nih.gov/?term=de+Roos+MAJ&cauthor_id=37679735)^11^, [Veerle M.H. Coupé](https://pubmed.ncbi.nlm.nih.gov/?term=Coup%C3%A9+VMH&cauthor_id=37679735)^12^, [Geraldine R. Vink](https://pubmed.ncbi.nlm.nih.gov/?term=Vink+GR&cauthor_id=37679735)^13^, [Cornelis Verhoef](https://pubmed.ncbi.nlm.nih.gov/?term=Verhoef+C&cauthor_id=37679735)^1^, Dirk J. Grünhagen^1^, [Johannes H.W. de Wilt](https://pubmed.ncbi.nlm.nih.gov/?term=de+Wilt+JHW&cauthor_id=37679735)^2^

^1^Department of Surgical Oncology and Gastro-Intestinal Surgery, Erasmus MC Cancer Institute, University Medical Centre Rotterdam, The Netherlands

^2^Department of Surgery, Radboud University Medical Centre, Nijmegen, The Netherlands.

^3^Department for Health Evidence, Radboud University Medical Centre, Nijmegen, The Netherlands.

^4^Department of Medical Psychology, Radboud University Medical Centre, Nijmegen, The Netherlands.

^5^Department of Research and Development, Netherlands Comprehensive Cancer Organisation, Utrecht, The Netherlands.

^6^Department of Surgery, Bernhoven Hospital, Uden, The Netherlands.

^7^Department of Surgery, Catharina Hospital, Eindhoven, The Netherlands.

^8^Department of Surgical Oncology, University Medical Centre Utrecht, Utrecht University, Utrecht, The Netherlands.

^9^Department of Surgery, Gelre Hospitals, Apeldoorn, The Netherlands.

^10^Department of Surgery, Jeroen Bosch Hospital, 's-Hertogenbosch, The Netherlands.

^11^Department of Surgery, Rijnstate Hospital, Arnhem, The Netherlands.

^12^Department of Epidemiology and Data Science Amsterdam Public Health, Amsterdam UMC, Vrije Universiteit Amsterdam, Amsterdam, The Netherlands.

^13^Department of Medical Oncology, University Medical Centre Utrecht, Utrecht University, Utrecht, The Netherlands.

**Corresponding author:**

M.H.E. van Driel, MD

Department of Surgical Oncology and Gastrointestinal Surgery, Erasmus MC Cancer Institute, Rotterdam

Dr. Molewaterplein 40, 3015 GD Rotterdam

Email [m.h.e.vandriel@erasmusmc.nl](mailto:m.h.e.vandriel@erasmusmc.nl) or telephone +31 010 7042125

**Supplementary Materials - Index**

| **Supplementary Appendixes** |  |
| --- | --- |
| Overview of the actual timing of implementation in the stepped-wedge cluster randomised trial | Pag. 3 |
|  |  |
| Overview of the initially planned stepped-wedge design and intended implementation schedule across participating hospitals | Pag. 3 |
|  |  |
| Overview of screening per hospital - numbers included, numbers excluded and reasons for exclusion | Pag. 4 |

**Supplementary Appendixes**

**Appendix A. Actual timing of implementation in the stepped-wedge cluster randomised trial**

|  | July '22 | Aug '22 | Sept '22 | Oct '22 | Nov '22 | Dec '22 | Jan '23 | Feb '23 | Mar '23 | Apr '23 | May '23 | June '23 | July '23 | Aug '23 | Sept '23 | Oct '23 | Nov '23 | Dec '23 | Jan '24 | Feb '24 | Mar '24 | Apr '24 | May '24 |
| --- | --- | --- | --- | --- | --- | --- | --- | --- | --- | --- | --- | --- | --- | --- | --- | --- | --- | --- | --- | --- | --- | --- | --- |
| **Centre** |  |  |  |  |  |  |  |  |  |  |  |  |  |  |  |  |  |  |  |  |  |  |  |
| **1** | * |  |  |  |  |  |  |  |  |  |  |  |  |  |  |  |  |  |  |  |  |  |  |
| **2** |  |  |  |  |  |  |  |  |  |  |  |  |  |  |  |  |  |  |  |  |  |  |  |
| **3** | * |  |  |  |  |  |  |  |  |  |  |  |  |  |  |  |  |  |  |  |  |  |  |
| **4** |  |  |  |  |  |  |  |  |  |  |  |  |  |  |  |  |  |  |  |  |  |  |  |
| **5** | * |  |  |  |  |  |  |  |  |  |  |  |  |  |  |  |  |  |  |  |  |  |  |
| **6** |  |  |  |  |  |  |  |  |  |  |  |  |  |  |  |  |  |  |  |  |  |  |  |
|  |  |  |  |  |  |  |  |  |  |  |  |  |  |  |  |  |  |  |  |  |  |  |  |
|  | Including patients in standard FU | | | | | | | | | | | | | | | | | | | | | |  |
|  | Including patients in PHFU = patient-led, home-based follow-up | | | | | | | | | | | | | | | | | | | | | |  |
| * | Study not initiated yet | | | | | | |  |  |  |  |  |  |  |  |  |  |  |  |  |  |  |  |

**Appendix B. Planned stepped-wedge design and intended implementation schedule across participating hospitals**

|  | July '22 | Aug '22 | Sept '22 | Oct '22 | Nov '22 | Dec '22 | Jan '23 | Feb '23 | Mar '23 | Apr '23 | May '23 | June '23 | July '23 | Aug '23 | Sept '23 | Oct '23 | Nov '23 | Dec '23 | Jan '24 |
| --- | --- | --- | --- | --- | --- | --- | --- | --- | --- | --- | --- | --- | --- | --- | --- | --- | --- | --- | --- |
| **Centre** |  |  |  |  |  |  |  |  |  |  |  |  |  |  |  |  |  |  |  |
| **1** |  |  |  |  |  |  |  |  |  |  |  |  |  |  |  |  |  |  |  |
| **2** |  |  |  |  |  |  |  |  |  |  |  |  |  |  |  |  |  |  |  |
| **3** |  |  |  |  |  |  |  |  |  |  |  |  |  |  |  |  |  |  |  |
| **4** |  |  |  |  |  |  |  |  |  |  |  |  |  |  |  |  |  |  |  |
| **5** |  |  |  |  |  |  |  |  |  |  |  |  |  |  |  |  |  |  |  |
| **6** |  |  |  |  |  |  |  |  |  |  |  |  |  |  |  |  |  |  |  |
|  |  |  |  |  |  |  |  |  |  |  |  |  |  |  |  |  |  |  |  |
|  | Including patients in standard FU | | | | | | | | | | | | | | | | | | |
|  | Including patients in PHFU = patient-led, home-based follow-up | | | | | | | | | | | | | | | | | | |

**Appendix C Overview of screening per hospital - numbers included, numbers excluded and reasons for exclusion**

|  | Hospital 1 | Hospital 2 | Hospital 3 | Hospital 4 | Hospital 5 | Hospital 6 | Total |
| --- | --- | --- | --- | --- | --- | --- | --- |
| Number screened | 153 | 313 | 91 | 140 | 109 | 357 | 1163 |
| Included | 78 | 85 | 46 | 70 | 42 | 45 | 366 |
| Excluded | 75 | 228 | 45 | 70 | 67 | 312 | 797 |
| Wrong tumour stage (T1, T4, benign) | 26 | 57 | 9 | 35 | 41 | 110 | 278 |
| No (regular) FU* | 16 | 34 | 7 | 9 | 3 | 144 | 213 |
| Recurrent disease | 12 | 48 | 15 | 8 | 13 | 21 | 117 |
| Not surgically treated | 8 | 15 | 8 | 8 | 4 | 15 | 58 |
| Other study / trial | 5 | 10 | 1 | 2 | 1 | 1 | 20 |
| Other malignancy | 3 | 10 | 1 | 2 | 1 | 17 | 34 |
| Medical complications | 2 | 3 | 1 | 2 | 3 | 2 | 13 |
| Deceased | 1 | 1 | 1 | 3 | 1 | 2 | 9 |
| Unknown | 2 | 50 | 2 | 1 | 0 | 0 | 55 |

** No (regular) follow-up entailed follow-up in a different hospital, and/or reduced or no follow-up due to old age*
