# Supplementary material for: Patient-led, home-based follow-up for colorectal cancer: DISTANCE multicentre stepped-wedge cluster randomised trial
Source: Br J Surg. 2026 Jun 26;113(8):znag081. doi: 10.1093/bjs/znag081 (PMC13454712; doi:10.1093/bjs/znag081)
Supplement: Supplementary file 1 [file znag081_supplementary_data.zip › CONSORT DISTANCE filled in template 20-04-2026.doc]

**BJS CONSORT diagram template**

Assessed for eligibility

*n = 1163*

Randomized

*n* = 366

Excluded *n = 797*

Did not meet inclusion criteria *n = 742*

Other (unknown reason) *n = 55*

Allocated to control *n = 184*

Received control *n = 282*

Did not receive control *n =* *19*

*Received no FU n = 1*

*Withdrawal of IC PLCRC n = 2*

*Wrong staging before inclusion n = 6*

*Crossover to intervention group n = 10*

Allocated to intervention *n = 182*

Received intervention *n = 72*

Did not receive intervention *n = 120*

*Received no FU = 2*

*Withdrawal of IC PLCRC n = 1*

*Crossover to control group n = 117**

Lost to follow-up *n =* 0

Give reasons *n* =

Discontinued intervention *n* = 0

Give reasons *n =*

Lost to follow-up *n =* 0

Give reasons *n* =

Discontinued intervention *n* = 0

Give reasons *n =*

Analysed *n = 282*

Excluded from analysis *n = 0*

Analysed *n = 72*

Excluded from analysis *n = 0*

Analysis

Enrolment

Allocation

Follow-up

* Crossover occurred before start of the intervention or control follow-up within the study timeframe.
